# Survival benefit of surgery with radiotherapy vs surgery alone to patients with T2-3N0M0 stage esophageal adenocarcinoma

**DOI:** 10.18632/oncotarget.7256

**Published:** 2016-02-08

**Authors:** Yaqi Song, Guangzhou Tao, Qing Guo, Xi Yang, Hongcheng Zhu, Wanwei Wang, Xinchen Sun

**Affiliations:** ^1^ Department of Radiation Oncology, Huai'an First People's Hospital, Nanjing Medical University, Huai'an 223300, China; ^2^ Department of Radiation Oncology, The First Affiliated Hospital of Nanjing Medical University, Nanjing 210029, China; ^3^ Department of Oncology, Taizhou people's hospital, Taizhou 225300, China

**Keywords:** esophageal cancer, adenocarcinoma, radiotherapy, surgery, SEER program

## Abstract

**Background & Aims:**

This study is designed to analyze survival benefit of (neo-) adjuvant radiotherapy to patients with T2-3N0M0 stage esophageal adenocarcinoma (EAC).

**Methods:**

T2-3N0M0 stage EAC patients from 2004 to 2012 were searched from the Surveillance Epidemiology and End Results (SEER) data. Clinical factors including age, sex, race were summarized. Univariate, multivariate analysis, and stratified cox analysis based on different T stages were performed to explore the survival effect of (neo-)adjuvant radiotherapy to T2-3N0M0 stage EAC.

**Results:**

T2-3N0M0 stage EAC patients with surgery were more likely to be white race, T3 stage. Univariate analysis showed sex, age, and T stage were the prognostic factors of survival (P<0.05). Multivariate analysis proved (neo-)adjuvant radiotherapy can prolong survival time of T2-3N0M0 stage EAC (P<0.05). Further analysis based on different T stages showed that both neoadjuvant radiotherapy (HR 0.615; 95% CI 0.475-0.797) and adjuvant radiotherapy (HR 0.597; 95% 0.387-0.921) significantly reduced the risk of death of T3N0M0 stage EAC, but neither of which significantly reduced death risk of T2N0M0 stage EAC (P>0.05).

**Conclusions:**

sex, age are the independent prognostic factors of T2-3N0M0 EAC. Significant survival benefit of (neo-)adjuvant radiotherapy is only observed in patients with T3N0M0 stage EAC, but not in those with T2N0M0 stage.

## INTRODUCTION

Esophagus cancer is the world's eighth incidence and the sixth cause of death cancer. 455,800 new diagnostic esophageal cancers and 400,200 deaths were reported to occur worldwide in 2012 [1]. Esophageal adenocarcinoma (EAC) is the most common esophageal malignancy. During the past decades, Treatment strategy of it has varied from surgery alone to multimodal approach [2–4]. Currently, surgery is still an irreplaceable treatment in localized stage EAC [5], but whether neoadjuvant or adjuvant radiotherapy could improve the survival of patients in early localized stage EAC is not clear [6] [7].

The Surveillance, Epidemiology, and End Results (SEER) Program is a cancer related database founded by the National Cancer Institute (NCI) in the United States. It collects and reports cancer incidence and survival data from population-based cancer registries and covers approximately 28% of the US population. With large information of cancer, it is an important tool to analyze carcinoma.

In view of above, we used SEER data for the analysis of EAC. Purpose to explore the efficacy of (neo-)adjuvant radiotherapy to the T2-3N0M0 stage EAC.

## RESULTS

A total of 918 patients were selected from the SEER database. In which, 338(36.8%) cases received surgery alone, 492(53.6%) cases received neoadjuvant radiotherapy with surgery (RT + Surg), and 88(9.6%) patients received surgery with adjuvant radiotherapy (Surg + RT). More than 95% patients were white race, so we combined black race in to other. Percent of male was 88.5%, much more than female. Independence test of the patients' treatment assignment and clinical characteristics indicated an obvious association of (neo-)adjuvant radiotherapy to age and T stage. Patients younger than 65 years and with the T3N0M0 stage were more likely to receive RT + Surg. A detailed listing of the patient characteristics and pathological features was presented in Table 1.

**Table 1:** Summary of characteristics and characteristics stratified by treatment

Variable		All patients(%)	Surg	RT+Surg	Surg+RT	χ^2^	P-valueψ
Sex	Female	106(11.5)	44	53	9	1.155	0.5613
	Male	812(88.5)	89	107	21		
Race	White	878(95.6)	320	474	84	1.344	0.511
	Other	40(4.4)	18	18	4		
T Stage	T2	367(40.0)	183	151	33	46.168	0.000***
	T3	551(60.0)	155	341	55		
Age	65−	454(50.5)	138	269	47	15.976	0.000***
	65+	464(49.5)	200	223	41		
	Surg+RT	88(9.6)	-	-	88		
radiotherapy	Surg	338(36.8)	338	-	-	-	-
	RT+Surg	492(53.6)	-	492	-		

Abbreviations: RT+Surg = neoadjuvant radiotherapy+surgery; Surg+RT = surgery + adjuvant radiotherapy ψ chi-square test

**Figure 1:**
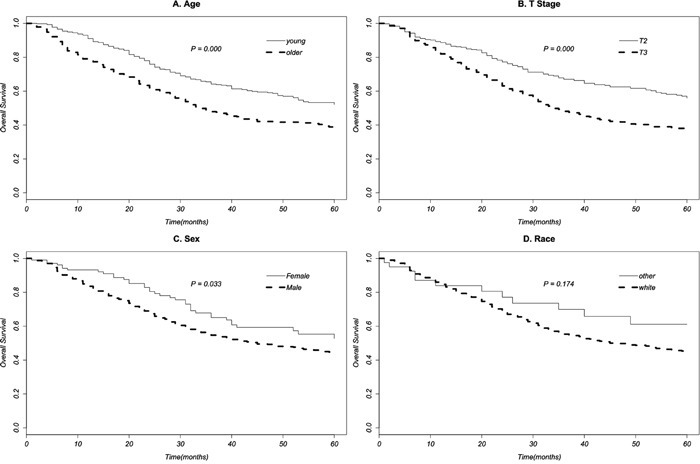
Survival curves of age A. T stage B. sex C. race D. to patients of T2-3N0M0 stage EAC

Univariate survival analysis of clinical characteristics was evaluated with log-rank test (Table 2). Age (Figure 1A), T stage (Figure 1B), and sex (Figure 1C) were significantly associated with survival time (P<0.05). Race (Figure 1D) showed no significant association with survival (P>0.05). Multivariate analysis performed with the Cox regression model showed age, T stage and (neo-)adjuvant radiotherapy were the independent prognostic factors of survival time (P<0.05). Young patients with T2N0M0 stage might have a longer survival time, both neoadjuvant and adjuvant radiotherapy prolonged survival time (Table 3).

**Table 2:** Univariate survival analysis of EAC patients

Variable	Univariate analysis	
	χ^2^	P-value^†^
**Sex**	4.6	0.033*
**T Stage**	27.3	0.000***
**Age**	15.6	0.000***
**Race**	1.8	0.174
**Radiation**	4.3	0.116

† Log–rank test.

**Table 3:** Multivariate cox proportional hazards regression analysis of EAC patients

Variable	Multivariate analysis	
	HR(95% CI)	P-value^‡^
**Sex**		
Female Vs Male	0.723(0.515-1.015)	0.061
**T Stage**		
T2 Vs T3	0.514(0.411-0.641)	0.000***
**Age**		
65- Vs 65+	0.613(0.500-0.750)	0.000***
**Race**		
White Vs Other	1.450(0.816-2.579)	0.205
**Radiation**		
RT+Surg Vs Surg	0.729(0.586-0.907)	0.004**
Surg+RT Vs Surg	0.668(0.464-0.961)	0.030*

Abbreviations: RT+Surg = neoadjuvant radiotherapy+surgery; Surg+RT = surgery + adjuvant radiotherapy; HR = hazard ratio; CI = confidence interval.

‡ Cox regression model test.

**Figure 2:**
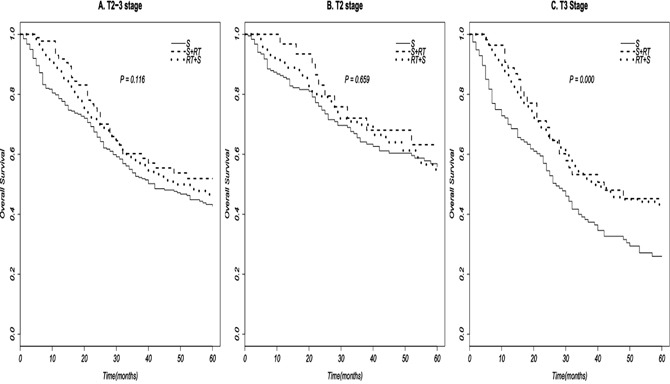
Survival curves of (neo-)adjuvant radiotherapy to EAC patient based on different T stages

Finally, we performed stratified multivariate cox regression analysis to assess the efficacy of (neo-) adjuvant radiotherapy to survival time based on different T stages, by adjusting for sex, race, and age (Table 4). The results displayed that compared with surgery alone, both RT+Surg (HR 0.615; 95% CI 0.475-0.797) and Surg+RT (HR 0.597; 95% CI 0.387-0.921) can significantly improve survival time of T3N0M0 stage EAC, but neither of which do significant survival benefit to T2N0M0 stage EAC. Survival curves of (neo-)adjuvant radiation therapy based on different T stages were in Figure 2.

**Table 4:** Multivariate cox proportional hazards regression analysis of radiotherapy based on different stages of EAC

Stage	OS	
	HR(95% CI)	P-value^‡^
**T2**		
RT+Surg Vs Surg	1.085(0.745-1.581)	0.671
Surg+RT Vs Surg	0.776(0.400-1.507)	0.454
**T3**		
RT+Surg Vs Surg	0.615(0.475-0.797)	0.000***
Surg+RT Vs Surg	0.597(0.387-0.921)	0.020*

Abbreviations: RT+Surg = neoadjuvant radiotherapy+surgery; Surg+RT = surgery + adjuvant radiotherapy; HR = hazard ratio; CI = confidence interval.

‡ Cox regression model test.

## DISCUSSION

Esophageal adenocarcinoma is one of the two major histological subtypes of esophageal cancer in the world, with a high and rapidly increased incidence in the western countries, including United States, Australia, France, and England [1, 8, 9]. Risk factors of EAC mainly contain gastroesophageal reflux disease (GORD), obesity, Barrett's oesophagus, tobacco use, and so on [10, 11]. Treatment scheme of EAC mainly includes surgery, chemotherapy, and radiotherapy [9]. Surgery is a principal locoregional treatment for patients, and widely performed in locally limited (cT1/T2, N0) and some resectable locally advanced carcinoma (cT3, T4, Nx) [3, 5]. Chemotherapy, as a systemic treatment, is commonly used in locally advanced, metastatic, and recurrent EAC [12–14], but less used in locally early stage [15]. Radiotherapy, as another important locoregional treatment, is usually used as a replacement and supplement of surgery [16–18], and believed to produce less survival benefit than surgery in early stage EAC [17, 19, 20]. But whether perioperative radiotherapy is benefit to locally early stage esophagus cancer is not clear [7]. Some studies proved radiotherapy before or after surgery benefited long-time survival [6, 21, 22], but others indicated no significant survival benefit of neoaduvant or adjuvant radiotherapy [20, 23].

In this study, we summarized the clinical characteristics of surgical patients with T2-3N0M0 stage EAC, with information provided by the SEER database from 2004 to 2012. Characteristics analyzed in our study contained sex, race, T stage, age, and radiation therapy. We found that operative patients with T2-3N0M0 EAC were more likely to be T3 stage (60.0%), male (88.5%), and white (95.6%) race. More than half of these EAC patients received additional radiation therapy (63.2%). Of which, neoadjuvant radiotherapy was 84.8% (53.6%/63.2%), and adjuvant radiotherapy was 15.2% (9.6%/63.2%). Independence chi-square test between radiation therapy and other factors showed that radiation therapy was associated with age and T stage. Young (<65 years) and T3 stage patients were more likely to receive neoadjuvant radiotherapy. Univariate survival analyses showed that survival time was associated with sex, age, and T stage (P<0.05), but of no association with race and radiotherapy (P>0.1). While multivariate cox proportional hazards regression analysis displayed that T stage, age, (neo-)adjuvant radiation therapy were all significantly associated with survival (P<0.05). Death risk of patients in T2N0M0 stage was lower than those in T3N0M0 stage (HR 0.514, 95% CI 0.411-0.641). Patients who younger than 65 years had a lower risk of death (HR 0.613, 95% CI 0.500-0.750). Adjuvant radiotherapy could reduce nearly 35% of death hazards (HR 0.668, 95% CI 0.464-0.961). And neoadjuvant radiotherapy could reduce nearly 30% of death hazards (HR 0.729, 95% CI 0.586-0.907). Further multivariate analysis of (neo-)adjuvant radiotherapy based on different T stages showed that both neoadjuvant radiotherapy and adjuvant radiotherapy could reduce more than 40% risk of death in T3 stage (P<0.05). But neither of them could significantly reduce death risk in T2 stage (P>0.05).

In conclusion, our study demonstrates that age and T stage are survival associated factors in T2-3N0M0 stage EAC. Compared with surgery alone, both neoadjuvant radiotherapy with surgery and surgery with adjuvant radiotherapy are of significant survival benefit to T3N0M0 stage EAC. But neither of them does significantly good to T2N0M0 stage of EAC.

## MATERIALS AND METHODS

### Patients

We chose SEER data between 1973 and 2012 [“Incidence - SEER 18 Regs Research Data + Hurricane Katrina Impacted Louisiana Cases, Nov 2014 Sub (1973-2012 varying)”] for this study. The National Cancer Institute's SEER*Stat software (Version 8.2.1) was used for the identity of patients. The inclusion criteria contained: (1) primary esophageal cancer (C15.0-C15.9) with a confirmed diagnosis of microscopically, (2) entire adenocarcinoma histology (Histologic/Behavior codes: 8140/3) based on the International Classification of Diseases for Oncology, 3rd Edition (ICD-O-3), (3) being diagnosed between 2004 and 2012, (4) with the 6th AJCC stage of T2-3N0M0, and (5) received surgery. And the exclusion criteria contained: (1) unknown age, sex, race, T, N, M stage, (2) with a radiotherapy status of “radiation both before and after surgery”, “intraoperative radiation therapy”, “intraoperative radiation with other radiation given before or after surgery”, “surgery both before and after radiation (for cases diagnosed 1/1/2012 and later)”, or “sequence unknown, but both surgery and radiation were given”, (3) diagnosed solely on autopsy or death certificate. Survival data were extracted at 1-month intervals for a maximal follow-up of 60 months.

This study based on public data from the SEER database. The reference number we obtained for the permission to access research data files was 10612-Nov2014. No human subjects or personal identifying information were used in this study. No informed consent was require in this study. This study was approved by the Review Board of Huai'an First People's Hospital, Huai'an, China.

### Statistical analysis

The enrolled population was divided into three groups based on different treatment: patients who were treated with surgery alone (Surg group), with surgery followed by radiotherapy (Surg+RT group), and with surgery following with radiotherapy (RT+Surg group). Chi-square test was used to analyze the differences of these three groups. Univariate analyses with log-rank test and multivariate analysis with cox proportional hazards regression model were performed to examine the clinical factors' association with survival respectively, with a statistically significant difference at the value of p<0.05. Finally, stratified cox regression survival analysis were performed based on different T stages. All analysis were performed with survival package [24] of R(version 3.2.1) [25].
